# Sex Steroids in COVID-19 Patients with Hypertension: An Exploratory Study

**DOI:** 10.3390/ijms26188976

**Published:** 2025-09-15

**Authors:** Pavitra Kotini-Shah, Shaveta Khosla, Felipe Borges Almeida, Luca Spiro Santovito, Heather Prendergast, Graziano Pinna

**Affiliations:** 1 Department of Emergency Medicine, College of Medicine, University of Illinois Chicago, 808 S. Wood St., MC 724, Chicago, IL 60612, USA; skhosl2@uic.edu (S.K.);; 2 The Psychiatric Institute, Department of Psychiatry, College of Medicine, University of Illinois Chicago, 1601 W. Taylor Street, Chicago, IL 60612, USA; felipeba@ufcspa.edu.br (F.B.A.);; 3 Graduate Program in Health Sciences, Federal University of Health Sciences of Porto Alegre (UFCSPA), Porto Alegre 90050-170, Brazil; 4 UI Center on Depression and Resilience (UICDR), Department of Psychiatry, College of Medicine, University of Illinois Chicago, Chicago, IL 60612, USA; 5 Center for Alcohol Research in Epigenetics (CARE), Department of Psychiatry, College of Medicine, University of Illinois Chicago, Chicago, IL 60612, USA

**Keywords:** COVID-19, sex steroids, estradiol, progesterone, testosterone, neuroactive steroids, hypertension, emergency department

## Abstract

Sex and gender disparities have emerged as critical determinants of COVID-19 outcomes, with males exhibiting higher hospitalization and mortality rates than females. Sex steroids such as estradiol, progesterone, and testosterone have been proposed as modulators of these differences, given their known roles in inflammation, immune function, and vascular health. However, the precise hormonal mechanisms underlying COVID-19 severity, particularly among individuals with comorbid hypertension—a major risk factor for adverse outcomes—remain unclear. In this study, we investigated circulating levels of key sex hormones and their neuroactive metabolites in 116 hypertensive COVID-19 patients enrolled through an urban academic emergency department. Our findings revealed distinct sex-based hormonal profiles and associations with disease severity. Males exhibited higher serum estradiol and testosterone levels, while progesterone levels were significantly higher in postmenopausal females. Notably, hospitalized patients showed elevated estradiol and progesterone levels compared to non-hospitalized individuals, whereas ICU-admitted patients had significantly lower concentrations of all three hormones. A unique exception was ICU-admitted postmenopausal females, who exhibited increased serum testosterone levels relative to non-ICU females. Additionally, in males, elevated 3α-diol was associated with hospitalization and ICU admission, while lower allopregnanolone and estradiol levels correlated with hypoxia in males and females, respectively. These results highlight a dynamic, sex-specific hormonal response to COVID-19 progression in hypertensive individuals, suggesting early upregulation and late depletion of protective sex steroids. Understanding these patterns may improve clinical risk stratification and inform the development of sex-targeted therapeutic interventions for COVID-19 and related inflammatory conditions.

## 1. Introduction

Since the beginning of the COVID-19 pandemic, sex and gender disparities have been evident with regard to disease prevalence, severity, and mortality [1,2,3,4]. Hospitalization and intensive care unit (ICU) admissions were reported to be higher in males than in females [5,6]. Scully et al. recently reported that the case fatality rate for males is 1.7 times higher than for females [7]. Despite these epidemiological data trends, the underlying molecular mechanisms and pathophysiology have remained unclear [3]. Preliminary speculations on the emerging role of androgens as a potential culprit in association with worse prognosis and female sex steroids as possibly protective factors [8,9,10,11]. In addition, the confluence of sex hormones, aging, and inflammation as closely connected aspects has also been investigated [12,13]. Consistently, several pathological conditions that are a risk factor for COVID-19 symptom severity, including metabolic syndrome and type 2 diabetes, are often observed in individuals who express lower blood testosterone levels [14,15,16]. Previous studies have also observed a correlation between COVID-19 symptom severity and respiratory intensive care unit (RICU) hospitalizations with decreased total and free testosterone blood concentrations [17]. Another study found a significant inverse correlation between baseline serum total testosterone concentration decrease and the percentage increase to be admitted to the ICU. Likewise, the total testosterone concentration decrease was significantly correlated with the % probability of mortality [17,18]. While these studies collectively suggest that decreased baseline levels of testosterone may predict COVID-19 symptom severity and mortality specifically in men admitted to the ICU, the role and the mechanisms involving sex hormones in COVID-19 illness remain largely elusive.

A major contributing factor to the differing mortality in COVID-19 in men versus women has also been seen in animal models. Male mice expressing SARS-CoV infection had higher mortality, and the lower mortality in female mice was attributed to the protective role of estrogen [19]. One of the major forms of estrogen, estradiol, has been shown to dampen the production of excessive innate inflammatory cytokines by monocytes and macrophages [19]. Most critically, the host cell entry receptor for SARS-CoV-2, the angiotensin-converting enzyme 2 (ACE2), is regulated by estrogen [11,20].

Among COVID-19 comorbidities and risk factors, high blood pressure, or hypertension, has been one of the most prevalent and critical in increasing the odds for hospitalization, ICU admission, and mortality [21]. Like for COVID-19, hypertension shows a strong sex dimorphism, which points to sex steroids as a possible underlying cause [22]. Indeed, experimental studies have observed that testosterone and estrogen play an important role in regulating blood pressure and hypertension. For example, estrogens may induce vasodilation, which results in lower blood pressure, whereas androgens have been reported to induce both vasoconstriction and vasorelaxation [23,24,25]. Some studies in women have observed higher testosterone and estradiol levels in individuals with hypertension [26,27,28]. However, studies that investigated the role of sex steroid levels in hypertension have generally originated mixed results. Intriguingly, the role of sex steroids in the underlying mechanisms contributing to COVID-19 symptom severity and mortality in individuals who suffer hypertension remains under-investigated.

Overall, with the understanding that male sex is a risk factor for developing severe SARS-CoV infection, in this investigation, we aimed to characterize the predominant sex hormones progesterone, estradiol, and testosterone in COVID-19 disease severity and progression in serum obtained from patients with hypertension. We also studied the serum concentrations of progesterone’s metabolites, including the isomers of allopregnanolone, epipregnanolone, pregnanolone, and isoallopregnanolone, and the metabolites of testosterone, including 3α-diol and 3β-diol. A better understanding of sex differences and the role of sex steroids in COVID-19 would help to optimize diagnostic strategies but also the proper pharmacological management of patients with COVID-19.

## 2. Results

### 2.1. Demographic Characteristics

The demographic and clinical outcomes of a total of 116 participants with hypertension enrolled through an urban academic emergency department are detailed in Table 1. The majority (55.6%) were non-Hispanic Black, 34.8% were Hispanic, 5.2% were non-Hispanic White, and the remaining patients were of other racial and ethnic groups. 54.3% of the sample was male, and 47% were ≥65 years of age (84.5% were ≥50 years of age). Only 20.1% of participants had private insurance, and 43.1% were on Medicare, 23.3% on Medicaid, and the remaining were either ‘Other’ insurance, no insurance, or unknown.

The majority of the participants were hospitalized (83.6%), and 28.4% were hospitalized for >7 days. The individuals who had been admitted to the ICU were 15.5%, and 4 of them had received ventilator support (Table 1). There were no differences in demographic characteristics and clinical outcomes by sex, as presented in Table 1.

Cardiovascular disease was present as a comorbidity in 27.6% of the sample, with a higher prevalence in males than females (*p* = 0.05), as shown in Table 2. Moreover, 17.2% (*n* = 20/116) of the sample had either a prior history of atrial fibrillation, atrial flutter, Atrial tachycardia, ventricular tachycardia (VT), ventricular fibrillation (Vfib), supraventricular tachycardia (SVT), or new onset arrhythmia, or both. Of these 20, almost all (*n* = 18/20) had atrial fibrillation/atrial flutter, and one was a new onset arrythmia.

### 2.2. Sex Steroid Levels in Male and Females with Hypertension and COVID-19 Infection

**Figure 1:**
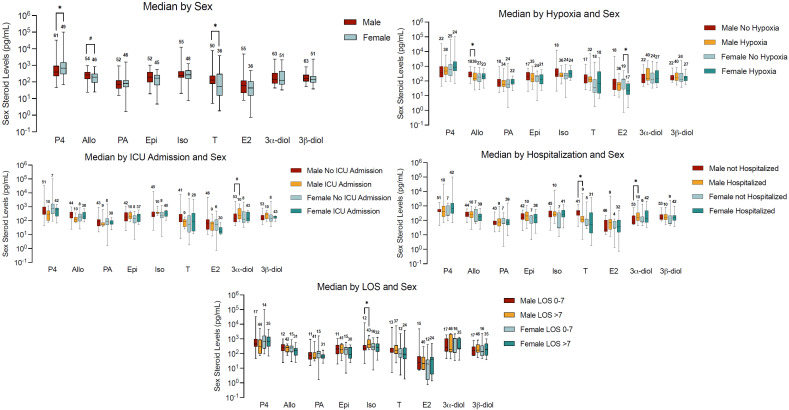
Distribution of hormones by sex (overall) and by sex and clinical outcomes. Median serum concentrations (pg/mL) of progesterone (P4), allopregnanolone (Allo), pregnanolone (PA), epipregnanolone (Epi), isoallopregnanolone (Iso), estradiol (E2), 3α-androstanediol (3α-diol), 3β-androstanediol (3β-diol), and testosterone (T) are shown across five subpanels: Top left: Median—by Sex: Progesterone levels were significantly higher in postmenopausal females than in males (*p* = 0.03), while testosterone (*p* = 0.01) and allopregnanolone (*p* = 0.066) were lower in females. No significant sex differences were observed for the other hormones. Top right: Median—by Hypoxia and Sex: Among males, hypoxia was associated with significantly lower serum allopregnanolone concentrations (*p* = 0.046). In females, hypoxia was associated with significantly lower estradiol levels (*p* = 0.013). Middle left: Median—by ICU Admission and Sex: In males, ICU admission was associated with marginally higher serum 3α-diol levels (*p* = 0.06). ICU patients of both sexes had generally lower progesterone, estradiol, and testosterone levels compared to non-ICU patients, except ICU females, who exhibited relatively higher testosterone than non-ICU females. Middle right: Median—by Hospitalization and Sex: Hospitalized males had lower testosterone (*p* = 0.0146) and higher 3α-diol (*p* = 0.048) levels than non-hospitalized males. Hospitalized females had the highest median progesterone levels compared to hospitalized males. Estradiol levels were higher in hospitalized males but lower in hospitalized females compared to their respective non-hospitalized counterparts. Bottom center: Median—by Length of Stay (LOS) and Sex: In males, serum isoallopregnanolone levels were significantly higher in those with a longer length of stay (>7 days; *p* = 0.016). These findings illustrate a sex-dimorphic and outcome-specific profile of circulating sex steroid concentrations in COVID-19 patients with hypertension, indicating possible prognostic and mechanistic relevance. Statistical comparisons were performed using the Mann–Whitney U test. All analyses were conducted in SAS version 9.4, with statistical significance defined as * *p* < 0.05. Marginally significant results are indicated with “#”. Data are presented as box-and-whisker plots showing the median, interquartile range (Q1–Q3), minimum and maximum values, and sample size (*n*).

Figure 1 shows that, as expected, progesterone levels in serum were higher in females (*p* = 0.03) than in males, while testosterone (*p* = 0.01) and allopregnanolone (*p* = 0.066) concentrations were lower both in terms of mean and median in females. No difference was observed with respect to other hormones.

### 2.3. Sex Steroid Levels and Clinical Outcomes in Subjects with Hypertension and COVID-19 Infection

Serum testosterone concentrations were much lower in COVID-19 hospitalized males with hypertension than in non-hospitalized males (*p* = 0.0146) (Figure 1). In contrast, the testosterone metabolite 3α-diol content was higher in hospitalized males when compared to non-hospitalized males (*p* = 0.048). No differences were observed in sex steroid levels among females by hospitalization status. Serum 3α-diol content was also higher in males that were admitted to the ICU than in males that were not admitted and was marginally significant (*p* = 0.06).

The progesterone metabolite, allopregnanolone, concentrations in serum were lower in males that were hypoxic than in males that were not hypoxic (*p* = 0.046). In females, serum estradiol content was much lower in those with hypoxia than those without hypoxia (*p* = 0.013).

In males, the progesterone metabolite, isoallopregnanolone serum content, was higher in those that had a longer length of stay (>7 days; *p* = 0.016).

### 2.4. Sex Steroid Concentrations and Symptoms in Male and Females with Hypertension and COVID-19 Infection

**Figure 2:**
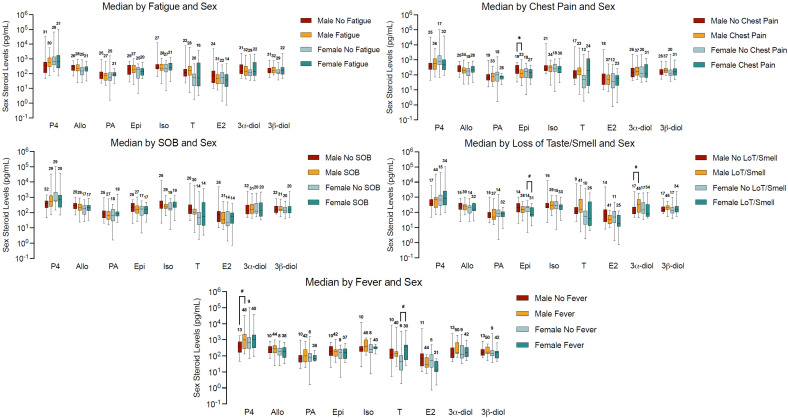
Distribution of sex steroid hormone concentrations by sex and symptom profile in hypertensive COVID-19 patients. Median serum concentrations (pg/mL) of the same nine sex steroids are presented across five panels, stratified by symptom presence and sex: Top left—median by fatigue and sex: no significant differences in hormone levels were found by fatigue status. Top right—median by chest pain and sex: In males, epipregnanolone levels were significantly lower in those with chest pain (*p* = 0.018); no significant differences in females. Middle left—median by shortness of breath (SOB) and sex: no statistically significant associations observed. Middle right—median by loss of taste (LOT)/smell and sex: in males, 3α-diol levels were higher in those reporting loss of smell/taste (*p* = 0.06); in females, epipregnanolone levels were lower in symptomatic individuals (*p* = 0.057). Bottom center—median by fever and sex: males with fever showed marginally higher progesterone (*p* = 0.05); in females, testosterone levels were marginally higher in febrile patients (*p* = 0.052). Statistical comparisons were performed using the Mann–Whitney U test. All analyses were conducted in SAS version 9.4, with statistical significance defined as * *p* < 0.05. Marginally significant results are indicated with “#”. Data are presented as box-and-whisker plots showing the median, interquartile range (Q1–Q3), and sample size (*n*).

We analyzed disease progression, defined by symptoms of hypoxia, shortness of breath, fever, loss of taste or smell, and fatigue, and found sex-stratified differences in hormone levels. Progesterone levels in males that had a fever were marginally significantly higher (*p* = 0.05) than in males who did not have a fever, while its metabolite, epipregnanolone, serum concentrations were lower among those that had chest pain (*p* = 0.018). In female patients who presented with fever, serum testosterone concentration also tended to be higher (*p* = 0.052). The testosterone metabolite, 3α-diol, concentrations in serum were higher for males who had loss of smell/taste, even though they did not reach significance (*p* = 0.06). The loss of smell/taste was associated with a tendency of lower concentration levels of epipregnanolone in serum (*p* = 0.057) in females (Figure 2).

### 2.5. Sex Dimorphism in Steroid Levels and Hospitalization and ICU Admission

When stratified by hospitalization and sex, serum progesterone levels did not differ by hospitalization or sex in patients with hypertension and COVID-19 infection. However, some differences were noted. Hospitalized females had generally lower serum estradiol concentrations, while hospitalized males had higher estradiol levels. In contrast, progesterone levels were higher in hospitalized females compared to hospitalized males, while hospitalized males had the highest estrogen levels compared to all three categories. Consistent with males having higher serum estradiol levels relative to females, male ICU patients had higher estradiol levels relative to female ICU patients. However, male ICU patients had lower estradiol levels compared to males not admitted to the ICU. Similarly, female ICU patients had significantly lower estradiol levels compared to females not admitted to the ICU. Serum progesterone concentration was lower in ICU males and females relative to males and females not admitted to the ICU.

In general, across all three main sex hormones examined (progesterone, estradiol, and testosterone), ICU patients of both sexes had lower levels of hormones compared to non-ICU patients, except for ICU females, who had higher serum testosterone concentrations compared to non-ICU females.

## 3. Discussion

This study was conducted in an ethnically diverse sample of 116 male and female patients with hypertension that contracted COVID-19 and were enrolled through an urban academic emergency department [29]. All females were above >50 years old and considered post-menopausal. Their disease severity was assessed by hospitalization, ICU admission, and vasopressor use. In our study, we describe for the first time sex hormone differences in COVID-19 patients. We show that in males there was a predominance of serum estradiol concentrations compared to postmenopausal females, whereas serum progesterone levels were higher in females relative to males. Testosterone levels in serum were higher in males than in postmenopausal women. There was a paradoxical response for serum progesterone and estradiol concentrations for hospitalization vs. ICU admission. Among those individuals who were hospitalized, serum progesterone and estrogen concentrations were highest compared to patients who were not hospitalized. In contrast, patients who had been admitted to the ICU had lower serum levels of progesterone, estradiol, and testosterone compared to patients not admitted to the ICU. Generally, considering the main sex hormones examined—progesterone, estradiol, and testosterone—subjects that were admitted to the ICU of both sexes showed lower levels of sex steroids when compared to non-ICU patients. The female patients who were admitted to the ICU who exhibited higher serum testosterone levels when compared to non-ICU females were exceptions. Possible explanations for these paradoxical findings in progesterone and estradiol, specifically, may involve the progression and severity of the disease in the context of the influence of inflammatory processes.

Our study showed a sex dimorphism in sex steroid levels and clinical outcomes, including hospitalization and ICU admission. For instance, serum estradiol levels were found to be lower in post-menopausal females with hypertension and COVID-19 infection who were hospitalized, while in males, estradiol concentrations in serum were higher. Similarly, male subjects who were admitted to the ICU had higher estradiol concentrations versus female ICU patients. Furthermore, females who were hospitalized showed the highest median serum progesterone levels when compared to hospitalized males.

These dynamic, sex-specific changes in circulating sex steroid levels in response to COVID-19, particularly in the context of comorbid hypertension, suggest a general trend toward early upregulation of protective hormones during milder disease, followed by depletion as symptom severity increases. Prior literature has also alluded to the concept of hypothalamic hypogonadism after critical illness [30,31].

In males, high estradiol levels may result from increased aromatization of testosterone, a process catalyzed by the enzyme aromatase, which is upregulated during inflammation and adiposity [32,33]. This conversion is relevant in the context of hypertensive males with COVID-19, a population often characterized by increased metabolic inflammation. Elevated testosterone levels in these patients may reflect a maladaptive or compensatory response [34]. While testosterone has been shown to exert both pro-inflammatory and anti-inflammatory effects [35], high levels have been linked to adverse outcomes in women with COVID-19 [36]. In contrast, estradiol is generally associated with anti-inflammatory actions, with studies suggesting it attenuates pro-inflammatory cytokines through inhibition of NF-κB signaling and reduction in IL-6 and TNF-α production. However, in some contexts, including postmenopausal females with comorbid hypertension, higher estradiol levels have been correlated with increased disease severity, suggesting a possible loss of protective effect [37,38,39]. Emerging data suggest that both low and high testosterone levels, depending on timing and disease stage, can be associated with poor prognosis in COVID-19 [36].

Postmenopausal women show a substantial reduction in estradiol production, yielding lower circulating estrogen levels compared to premenopausal women and often even to age-matched men. In an individual affected by COVID-19, the decline of endogenous estrogen may contribute to impaired immunomodulation and vascular repair mechanisms, potentially exacerbating outcomes in postmenopausal females, particularly those with underlying cardiovascular conditions such as hypertension.

Interestingly, the hospitalized females group showed the highest median progesterone levels. Progesterone has been shown to have anti-inflammatory and immunomodulatory effects, potentially offering some protective role during moderate disease, though this was not observed at the ICU level.

With respect to hypertension, previous studies have demonstrated that sex hormones influence blood pressure regulation through vascular tone, renal sodium handling, and inflammation [26,28]. Lower estradiol and progesterone levels post-menopause are associated with heightened cardiovascular risk, which may compound the severity of COVID-19 outcomes in this subgroup. In the context of COVID-19, our results are consistent with prior reports that link altered sex steroid levels with immune dysregulation and worse clinical trajectories.

COVID-19 and similar acute inflammatory states can suppress the hypothalamic-pituitary-gonadal axis and disrupt adrenal function. In our study, hospitalized patients displayed higher sex steroid levels than non-hospitalized individuals, which may reflect an early upregulation of steroidogenic pathways. In contrast, ICU patients showed a generalized decline, consistent with endocrine exhaustion. This biphasic response mirrors patterns seen in other forms of systemic stress, for example, in neuropsychiatric conditions, and underscores the dynamic hormonal regulation during disease progression [40,41,42,43].

The progesterone metabolite, allopregnanolone, a neuroactive steroid and a potent positive allosteric modulator of GABA_A_ receptors with well-established anti-inflammatory properties [44], was reduced in hypoxic males, suggesting lowered anti-inflammatory protective mechanisms at a critical phase of illness. Conversely, its stereoisomer, isoallopregnanolone levels were elevated in males with prolonged hospitalization; however, its dysregulated metabolism remains with unclear functional impact. Epipregnanolone, another allopregnanolone’s isomer, concentrations were lower in males reporting chest pain and trended lower in those with loss of smell/taste, though its functional significance remains less well defined.

The testosterone metabolite 3α-diol was elevated in hospitalized and ICU-admitted males when testosterone was decreased, possibly reflecting increased testosterone metabolism through 5α-reductase during systemic inflammation. Overall, these findings point to a disruption in the balance of neuroactive steroids and androgen metabolites during severe COVID-19 infection, with potential implications for inflammatory regulation, but further mechanistic studies are needed to clarify their role.

Although our analysis centered on estradiol, progesterone, and testosterone, other steroids, such as DHEA, cortisol, estrone, and aldosterone, are also likely to shape inflammatory response in COVID-19. Future studies incorporating a broader steroid profile could elucidate interrelated endocrine mechanisms and clarify potential biomarkers of disease severity.

This study has several limitations. First, the sample size was modest, and multiple comparisons increased the potential for type I error. Second, the absence of a healthy control group limits our ability to interpret hormone levels in the context of baseline and levels of changes in pathophysiological conditions aggravated by COVID-19. Future work should include healthy subjects as well as incorporate a broader steroid and neuroactive steroid panel to allow for more informative comparisons. Third, although all women were considered postmenopausal b age, we lacked detailed information regarding hormone replacement therapy or exact menopausal timing, which may influence hormone levels. While exogenous hormone exposure was not assessed in our study, the potential influence of medications or hormone replacement therapy cannot be excluded. This represents a relevant consideration for future research, particularly in postmenopausal women whose endogenous steroid levels are already diminished and may respond differently to acute physiological stress. Finally, while hormone measurements were performed on serum collected during emergency admission, the time since infection onset was variable and not systematically recorded.

Notwithstanding the limitations of this exploratory study, if replicated in a larger, multi-center, longitudinal cohort, the observed biphasic pattern of sex steroids, initial elevation followed by decline with worsening severity, could inform risk stratification. For example, monitoring estradiol or progesterone levels upon hospital admission might help identify patients at risk of progressing to critical illness. Additionally, sex-specific hormonal signatures could guide more personalized treatment approaches, particularly in the context of immunosuppressive therapies or anti-inflammatory interventions.

## 4. Methods

### 4.1. Study Population

Participants enrolled in this study were patients treated or admitted through an urban academic emergency department between August 2020 and April 2021 in Chicago, USA. Male participants had to be >21 years of age, and female participants had to be 50 years of age and above, with a documented history of hypertension, tested positive for SARS-CoV-2, consented to participate in the study, and had blood drawn. Those that were unable to verbalize comprehension of the study or had impaired decision-making were excluded from the study [29].

### 4.2. Gas Chromatography-Mass Spectrometry Neuroactive Steroid Assays

Serum samples were allowed to settle for at least 30 min and then were centrifuged for 10 min at 3000 rpm and 4 °C to remove clots. Serum was aliquoted into 0.5 mL cryovials and stored at −80 °C until the time of sex steroid assays. We measured levels of progesterone, testosterone, and estradiol, examining absolute levels of each (pg/mL). Extraction, high-performance liquid chromatography (HPLC), derivatization, and gas chromatography-mass spectrometry (GC/MS) quantification analyses of sex steroids were performed as previously described [45,46]. Samples were extracted in ethyl acetate and lyophilized.

Sex steroids of interest were then purified and separated using HPLC. Tritiated sex steroids (American Radiolabeled Chemicals, St. Louis, MO, USA) were added to monitor the HPLC retention profile, while deuterated internal standards consisting of 2 pmol of deuterium-labeled sex steroids (CDN Isotopes, Pointe-Claire, QC, and Steraloids, Newport, RI, USA) were used to allow quantification of the compound of interest and correct for procedural losses. Each steroid of interest was then derivatized in heptafluorobutyric acid (HFBA) (ThermoFisher, Waltham, MA, USA) for GC/MS. Mass spectrometry analysis was performed in the standard electron impact mode for progesterone, testosterone, and estradiol measurements. The quantity of each steroid of interest was calculated by dividing the area under the peak of the steroid in the sample by the area under the peak of the deuterated internal standard. Progesterone sensitivity is ~0.03 ng/mL with an intra-assay coefficient of variation (CV) of ~4%. The detection limit for each steroid is approximately 10 fmol/mL. Intra-assay CV for these sex steroids is <5%.

### 4.3. Analytic Methods

Univariate analysis was conducted to assess the distribution of sex steroids and the other demographic and clinical variables, including the sex steroid hormone levels. Measures of central tendency (including mean, standard deviation, median, interquartile range, etc.) were examined for continuous variables, and frequencies were assessed for categorical variables. In addition to this, missing values were examined. No imputation was performed. Bivariate analyses were conducted to assess the distribution of demographic (such as age and race/ethnicity) and clinical variables (self-reported symptoms experienced over the past 14 days prior to receiving care at the enrolling medical center, including chest pain/tightness, fatigue, etc., and clinical outcomes such as hospitalization, length of stay, and ICU admission) by sex. Cardiovascular disease history was also analyzed by sex. Chi-square test and Fisher’s exact test were used to assess these associations by sex.

We further analyzed the difference in sex steroid levels by demographic and clinical variables when stratified by sex. As the hormone levels were not normally distributed, we used the non-parametric Mann–Whitney U tests to assess differences in the level of hormones by sex and other clinical variables. A *p*-value of 0.05 was used for statistical significance. Analyses were performed using SAS 9.4 (SAS Institute, Cary, NC, USA).

## 5. Conclusions

Our study highlights significant sex differences in serum sex steroid levels among hypertensive patients with COVID-19 and their association with disease severity. Studying sex-specific hormonal influences in this patient population is novel and important, and further investigation in larger, longitudinal cohorts to validate these findings may help to inform future clinical approaches.

Elevated estradiol and progesterone in hospitalized patients—but decreased levels in ICU admissions—suggest a dynamic hormonal response to disease progression. These findings underscore the importance of considering sex steroids in the context of COVID-19 outcomes and support further investigation into their mechanistic and therapeutic roles in infection, inflammation, and critical illness.

## Figures and Tables

**Table 1:** Demographic characteristics and clinical outcomes in subjects with hypertension that were treated or admitted through an urban academic emergency department after contracting COVID-19 (*n* = 116).

Variables	Categories	Overall *n* = 116	Female *n* = 53	Male *n* = 63
Age; Mean (SD)		62.1 (13.1)	64.3 (10.1)	60.3 (15.0)
Age group	≥65	55 (47.4%)	28 (52.8%)	27 (42.9%)
	<65	61 (52.6%)	25 (47.2%)	36 (57.1%)
Sex	Male	63 (54.3%)	-	63 (100%)
	Female	53 (45.7%)	53 (100%)	-
Race/Ethnicity	NH-Black	64 (55.2%)	32 (60.4%)	32 (50.8%)
	Hispanic/Latino	40 (34.5%)	17 (32.1%)	23 (36.5%)
	NH-White	6 (5.2%)		
	Other *	6 (5.2%)		
Fever **	Yes	22 (19.0%)	9 (17.0%)	13 (20.6%)
	No	94 (81.0%)	44 (83.0%)	50 (79.4%)
Shortness of Breath	Yes	64 (55.2%)	32 (60.4%)	32 (50.8%)
	No	52 (44.8%)	21 (39.6%)	31 (49.2%)
Chest Pain/tightness	Yes	46 (39.7%)	20 (37.7%)	26 (41.3%)
	No	70 (60.3%)	33 (62.3%)	37 (58.7%)
Loss of smell/taste	Yes	35 (30.2%)	18 (34.0%)	17 (27.0%)
	No	81 (69.8%)	35 (66.0%)	46 (73.0%)
Fatigue	Yes	62 (53.4%)	31 (58.5%)	31 (49.2%)
	No	54 (46.6%)	22 (41.5%)	32 (50.8%)
Hospitalized	Yes	97 (83.6%)	44 (83.0%)	53 (84.1%)
	No	19 (16.4%)	9 (17.0%)	10 (15.9%)
ICU admit	Yes	18 (15.5%)	8 (15.1%)	10 (15.9%)
	No	98 (84.5%)	45 (84.9%)	53 (84.1%)
Length of Stay	>7 days	33 (28.4%)	16 (30.2%)	17 (27.0%)
	0–7 days	83 (71.6%)	37 (69.8%)	46 (73.0%)
Ventilator support	Yes	4 (3.5%)		
	No	111 (96.5%)		
Hypoxia	Yes	48 (41.7%)	26 (49.1%)	22 (35.5%)
	No	67 (58.3%)	27 (50.9%)	40 (64.5%)
Vasopressor use	Yes	6 (5.2%)		
	No	110 (94.8%)		

* Includes unknown/missing. ** Fever was self-reported and defined as measured fever above 100 degrees F in the past 14 days prior to receiving care at the enrolling medical center. Grayed boxes are for variables that had very small samples when stratified by sex.

**Table 2:** Cardiovascular disease (including prior history and new onset) in the sample.

	Overall	Female *n* = 53	Male *n* = 63
Cardiovascular disease			
Yes	32 (27.6%)	10 (18.9%)	22 (34.9%)
No	84 (72.4%)	43 (81.1%)	41 (65.1%)
Prior history of atrial fibrillation/atrial flutter/atrial tachycardia/VT/Vfib/SVT AND/OR new onset arrhythmia during index presentation			
Yes	20 (17.2%)	9 (17.0%)	11 (17.5%)
No	96 (82.8%)	44 (83.0%)	52 (82.5%)
Any QTc 500 during index presentation			
Yes	7 (6.0%)	1 (1.9%)	6 (9.5%)
No	109 (94.0%)	52 (98.1%)	57 (90.5%)
Elevated Cardiac Markers > 0.04			
Yes	18 (15.5%)	9 (17.0%)	9 (14.3%)
No	98 (84.5%)	44 (83.0%)	54 (85.7%)
^a^ Significant valve disease			
Yes	6 (5.2%)	1 (1.9%)	5 (7.9%)
No	110 (94.8%)	52 (98.1%)	58 (92.1%)

^a^ Aortic stenosis/aortic insufficiency/mitral valve stenosis/mitral valve regurgitation moderate or greater.

## Data Availability

The original contributions presented in this study are included in the article. Further inquiries can be directed to the corresponding author(s).
